# The Yin-Yang Property of Chinese Medicinal Herbs Relates to Chemical Composition but Not Anti-Oxidative Activity: An Illustration Using Spleen-Meridian Herbs

**DOI:** 10.3389/fphar.2018.01304

**Published:** 2018-11-15

**Authors:** Yun Huang, Ping Yao, Ka Wing Leung, Huaiyou Wang, Xiang Peng Kong, Long Wang, Tina Ting Xia Dong, Yicun Chen, Karl Wah Keung Tsim

**Affiliations:** ^1^Shenzhen Key Laboratory of Edible and Medicinal Bioresources, Shenzhen Research Institute, Shenzhen, China; ^2^Division of Life Science and Center for Chinese Medicine, The Hong Kong University of Science and Technology, Clear Water Bay, Hong Kong; ^3^Department of Pharmacology, Shantou University Medical College, Shantou, China

**Keywords:** Yin-Yang classification criterion, cold and hot properties, principle component analysis, chemical composition, anti-oxidative activity

## Abstract

“Yin-Yang” and “Five Elements” theories are the basis theories of Traditional Chinese Medicine (TCM). To probe and clarify the theoretical basis of these ancient Chinese wisdoms, extensive efforts have been taken, however, without a full success. In the classification of TCM herbs, hot, cold and neutral herbs are believed to possess distinct profile of chemical compositions of which the compounds should have different polarity and mass: this view provides a new perspective for further illustration. To understand the chemical properties of TCMs in the classification of “Yin-Yang” and “Five Elements,” 15 commonly used herbs attributed to spleen-meridian were selected for analyses. Chemically standardized water extracts, 50% ethanol extracts and 90% ethanol extracts were prepared and subjected to different analytic measurements. Principle component analysis (PCA) of full spectrum of HPLC, NMR and LC-MS of the extracts were established. The results revealed that the LC-MS profile showed a strong correlation with the “Yin-Yang” classification criterion. The Yang-stimulating herbs generally contain more compounds with lower molecular weight and less polar property. Additionally, a comprehensive anti-oxidative profiles of selected herbs were developed, and the results showed that its correlation with cold and hot properties of TCM, however, was rather low. Taken together, the “Yin-Yang” nature of TCM is closely related to the physical properties of the ingredients, such as polarity and molecular mass; while such classification has little correlation with anti-oxidative property. Therefore, the present results provide a new direction in probing the basic principle of TCM classification.

## Introduction

In the theoretical basis of TCM clinical application, “Yin-Yang” and “Five Elements” theories could further differentiate into secondary classification principles, e.g., four natures and five flavors, meridian tropism, floating and sinking (Li et al., 2008; Fu et al., 2009, 2015; Mohd et al., 2013; Qiu, 2015). Having a long history of clinical experience, these TCM theories are aiming to merge with each other and achieve unity gradually. As the “Yin-Yang” attribution is not only specified to the description of patient symptoms (“Zheng” or syndrome), but also defines the nature of herbal medicines. Thus, the exact relationship between these two identification systems of TCM, i.e., “Yin-Yang” versus chemical composition, remains as a puzzle (Zhou et al., 2009; Ma et al., 2010; Li, 2012). In order to maintain a homeostasis in our body, the herbs with cold nature, also known as Yin-stimulating herbs, were generally used to treat hot diseases like inflammation, while the herbs with nature of warm and hot, belonging to Yang-stimulating herbs, were good for various types of deficiency (Fu et al., 2017a). In recent years, the works on chemical properties of TCMs has made a significant progress, and new ideas have been proposed, e.g., molecular drug hypothesis (Fu et al., 2017b), biodynamics (Yang et al., 2017), genetic hypothesis (Rezapour-Firouzi, 2017), transient receptor potential channel hypothesis (Bishnoi et al., 2018). Indeed, many of these newly proposed methods have been adopted and improved, including micro-calorimetry (Gao et al., 2014), mathematical modeling (Ramirez-Rodrigues et al., 2011), biophoton detection (Han et al., 2011; Zhao and Han, 2013). These methods are mainly derived from the collation of ancient books (Wang et al., 2010), pharmacological effects (Liang et al., 1988; Xu et al., 2011; Xiong et al., 2011), structure of components (Wang and Zhou, 2006) and thermodynamics (Yang et al., 2010). However, these studies are still lacking a co-relationship of the current TCM theory with the chemical composition of different classes of herbs.

Having highly developed analytical techniques and various novel chemometrics methods today, the study on relationship between TCM herb properties with their chemical composition has become possible. The widely employed analytical techniques, including HPLC, NMR and LC-MS, enabled researchers to establish robust methods to qualitative and quantitative determination of almost all kinds of compounds in biological matrices (Kuang et al., 2012). According to clinical application and experimental basis, the major ingredients corresponding to property and taste of TCM herbs are expected to be different. For example, volatile oil and polysaccharide are major bioactive components of pungent and sweet herbs; while alkaloid, glycoside and phenolic acid are generally abundant in bitter and acid herbs (Ung et al., 2007). In addressing these problems being encountered in TCM research, the omics techniques play a key role due to its advantage in solving information-rich puzzles and providing comprehensive and holistic results (Wiklund et al., 2008). Among the most commonly used multivariate statistical and visualization tools, principal component analysis (PCA) is a bilinear decomposition method that reduces original data to a few principal components and reserves the features contributing to variance (Kuang et al., 2012).

According to the concept of “Yin-Yang” nature of herbal medicine, the property of Chinese medicinal herbs was mainly summarized based on the efficacy and cognitive experience gained from patients’ response. Some pharmacological studies supported the notion that cold and hot properties of TCM herbs were closely related to excitability of nervous system and endocrine (Li et al., 2008; Fu et al., 2009), mitochondrial ATP generation, and immunomodulatory function (Ko et al., 2004, 2006; Ko and Leung, 2007). On the other hand, the potential relationship between “Yin-Yang” properties and redox system of herbs have been proposed (Ou et al., 2003). Nevertheless, the physical meaning behind the criteria of “Yin-Yang” classification is still missing (Szeto and Benzie, 2006; Wong et al., 2006). Moreover, previous research has mainly focused on chemically based of anti-oxidative activity in TCM herbs; however, the relationship of anti-oxidative property with chemical nature of herbs is still missing. PCA analysis of full spectrum of HPLC, NMR and LC-MS of different classes of herbs were conducted to provide a multi-angle and multi-dimensional perspectives of the connection between the “Yin-Yang” properties and their chemical composition. In addition, the possible relationship between “Yin-Yang” nature and anti-oxidant of various herbs was clarified.

## Materials and Methods

### Chemicals and Preparation of Herbal Extracts

HPLC-grade acetonitrile and ethanol were from Merck (Darmstadt, Germany). Ultra-pure water was obtained from a Milli-Q purification system (Millipore, Molsheim, France). Deuterium oxide (D_2_O), 3-(4,5-dimethylthiazol-2-yl)-2,5-diphenyltetra-zolium bromide (MTT), gallic acid, 1,1-Diphenyl-2-picrylhydrazyl radical 2,2-diphenyl-1-(2,4,6-trinitrophenyl) hydrazyl (DPPH), *tert*-butyl hydroperoxide (tBHP), vitamin C, 2′,7′-dichlorodihydrofluorescein diacetate (DCFH-DA), *β*-galactosidase, dimethyl sulfoxide (DMSO) and Hank’s Balanced Salt Solution (HBSS) were purchased from Sigma-Aldrich (St. Louis, MO, United States). Lipofectamine 3,000 was bought from Invitrogen (Waltham, MA, United States) and Triton X-100 was from BDH Tech (Marshalltown, IA, United States). The pARE-Luc DNA construct was obtained from Promega (Madison, WI, United States).

Three batches of 15 spleen-meridian herbs (Table 1) and a batch of Quinquefolium Radix, Rehmanniae Radix and Rehmanniae Radix Praeparata were collected from their production sites or pharmacy. The collected samples were first authenticated by Dr. Tina Dong and stored in dry and clean container before further analysis. The authentication of these herbs was according to Hong Kong Materia Medica Standards including morphology, microscopy, chemical fingerprint and chemical assay. The voucher specimens were stored in Centre for Chinese Medicine R&D at the university.

**Table 1:** The pharmacological action, origin and extracting yields of the tested 15 spleen-meridian herbs.

Attributes	Herbs	Major Composition	Actions	Origin/Drugstore	Yield (%, W/E50/E90)^a^
Yang herbs (hot, warm)	Codonopsis Radix	Polysaccharide, saponins, volatile oils, alkaloids, etc	To fortify spleen and replenish lung, **nourish blood**^b^	Gansu	27.8/23.6/18.5
				Gansu	23.6/24.1/16.4
				Gansu	22.2/21.8/14.6
	Atractylodis Macrocephalae Rhizoma	Volatile oil, saponins, polysaccharide, etc.	To fortify spleen and **check diarrhea**	Hengxing Pharmacy	12.8/9.77/11.7
				Zhejiang	14.7/13.1/16.7
				Yong Sheng Hang	17.4/12.4/17.2
	Angelicae Sinensis Radix	Volatile oil, flavonoids, organic acids, polysaccharide, amino acids, etc.	To **nourish** and active **blood**	Gansu	16.5/23.7/26.2
				Gansu	18.6/27.6/25.2
				Gansu	17.5/31.1/27.1
	Astragali Radix	Saponins, flavonoids, polysaccharide, etc.	To tonify qi, uprise yang and **nourish blood**	Shanxi	25.2/17.5/14.3
				Gansu	25.9/15.2/15.3
				Gansu	26.1/15.8/15.4
	Jujubae Fructus	Nutrients, flavonoids and phenolic acids, triterpenic acids, etc.	To tonify the middle energizer, **nourish blood**	Shandong	4.75/4.34/3.56
				Henan	4.78/3.66/3.33
				Ningxia	5.66/3.54/2.85
	Ginseng Radix	Ginsenosides, polysaccharide, volatile oils, etc.	To **tonify** the original **qi** and tonify spleen	Jilin	16.8/15.7/8.42
				Rong Chang Hang	15.8/14.6/7.38
				Jilin	14.8/13.2/8.38
	Amomi Fructus	Volatile oil, saponins, flavonoid glycosides, organic acid, etc.	Warm the spleen to **check diarrhea**	Hengxing Pharmacy	1.22/1.28/2.41
				Guangxi	1.32/1.22/2.53
				Yong Sheng Hang	1.04/1.21/1.91
	Aucklandiae Radix	Volatile oil, sesquiterpenes, amino acids, etc.	To move qi and **promote digestion**	Hengxing Pharmacy	1.15/2.45/5.44
				Sichuan	1.21/2.13/4.11
				Sichuan	1.17/2.02/3.02
	Areca Catechu	Alkaloids, flavonoids, tannins, triterpenes and steroids, fatty acids, etc.	To move qi and **check diarrhea**	Hengxing Pharmacy	3.04/3.89/4.24
				Hainan	2.43/3.33/4.51
				Yong Sheng Hang	1.49/2.48/4.42
Neutral herbs (Plain)	Dioscoreae Ahizoma	Polysaccharide, Saponins, etc.	To tonify spleen and stomach, **check diarrhea**	Hengxing Pharmacy	7.46/8.38/5.36
				Henan	9.49/7.43/7.45
				Henan	8.76/5.78/5.33
	Lablab Semen Album	Volatile oil, phytic acid, polyphenols, tannins, etc.	To fortify spleen, **check diarrhea** and resolve dampness	Hengxing Pharmacy	4.65/3.76/3.13
				Hunan	5.68/3.88/6.87
				Yong Sheng Hang	4.93/3.99/5.49
	Poria Cocos	Polysaccharide, triterpenoids, etc.	To fortify spleen and **calm the heart**	Hengxing Pharmacy	1.33/1.23/1.01
				Yunnan	1.32/1.34/1.11
				Yong Sheng Hang	1.42/1.46/1.31
Yin herbs (cool, cold)	Nelumbinis Semen	Alkaloids, flavonoids, amino acids, fatty acid, etc.	To tonify spleen, **check diarrhea** and **calm the heart**	Hengxing Pharmacy	5.65/4.33/3.22
				Jiangxi	5.54/3.56/2.32
				Hunan	6.86/4.55/3.58
	Coicis Semen	Esters, triglycerides, polysaccharide, sterols, alkaloids, etc.	Fortify the spleen to **check diarrhea**	Hengxing Pharmacy	2.64/1.51/0.49
				Hunan	3.32/1.67/1.11
				Yong Sheng Hang	2.63/1.21/1.01
	Crataegi Fructus	Flavonoids, triterpenoids, etc.	To **promote digestion** and invigorate the stomach, move qi	Hengxing Pharmacy	12.8/9.24/13.7
				Guangdong	11.5/7.46/13.2
				Shandong	16.5/6.65/15.3


*^a^W, water extract; E50, 50% ethanol extract; E90, 90% ethanol extract. ^b^Pharmacological effects were based on the Chinese Pharmacopoeia. The bold words are to emphasize the main pharmacological effects of the TCM.*

A unified extraction method was developed. Water extract, 50% ethanol extract and 90% ethanol extract of each herb were prepared to obtain extracts having different polarities. The specific extraction procedure was as following. First, the herbal samples were milled and passed through a No. 2 sieve and mixed well. Then, 4 g of the powdered sample was extracted in 100 mL distilled water, 50% ethanol and 90% ethanol, respectively, for 2 h by reflux, and the herbs were extracted twice. For the second extraction, the residue from the first extraction was filtered, and the same extraction condition was applied onto the filtered residue. Then, the extracts were combined, dried under vacuum and stored at 4°C.

The preparation of wine-treated Angelica Sinensis Radix was according to previous research (Zhan et al., 2011). To be specific, 30 g of the dried roots were sliced and sprayed with 3 mL of 15% ethanol and then processed in an oven at 80°C for 90 min with 3 flipping. Then 4 g of processed Angelica Sinensis Radix was weighed and prepared as the extraction procedure mentioned above.

### HPLC Fingerprint Analysis of Herbal Extracts

The fingerprint analysis of herbal extracts was performed on an Agilent HPLC 1200 series system (Agilent, Waldbronn, Germany), which was equipped with a degasser, a binary pump, an auto sampler, a thermostated column compartment and a DAD. The samples were separated on a Waters Symmetry C18 column (4.5 mm × 250 mm, 5 μm i.d.) after filtered with a guard column. The mobile phase was composed of acetonitrile (A) and water (B) according to the pre-set gradient program: 0–50 min, linear gradient 0–70% (A); 50–55 min, linear gradient 70–100% (A); 55–60 min, isocratic elution. A pre-balance period of 5 min was used between each run. The injection volume was 10 μL, and the flow rate was set at 1 mL/min. To get the fingerprints of herbal extracts, the wavelength of UV detector was set to 254 nm with full spectral scanning from 190 to 400 nm.

### NMR Spectrum Profiling

The^1^H NMR experiments were conducted to the extracts in revealing high abundance chemicals. Fifty mg of the dried extract was dissolved in 400 μL of D_2_O. All particulate materials were removed by centrifugation at 13,000 ×*g* for 1 min, and the supernatant was transferred to a standard 5-mm NMR tube. NMR spectra were acquired on a Varian 300 MHz NMR spectrometer, operating at 300.13 MHz ^1^H NMR frequency at 298 K. Gradient shimming was used to improve the magnetic field homogeneity prior to all acquisition. ^1^H NMR spectra of the samples were acquired using a 1D CPMG pulse sequence (RD-90°-t1-90°-tm- 90°-acquire) to generate a spectrum with a reduced residual solvent peak. The experiment time for each sample was around 10 min.

### LC-MS Spectrum Profiling

The LC-MS profiles of extracts were established on the Xevo G2-XS^®^ Quadrupole Time-of-Flight (Q-TOF) Mass Spectrometer coupled with an integrated ACQUITY UPLC^®^ I-Class System. The analysis column is ACQUITY UPLC^®^ BEH C18 column (2.1 mm × 50 mm, 1.7 μm). The mobile phase condition was recorded as follows: acetonitrile (A) and water (B), 0–0.5 min, isocratic gradient 5% (A); 0.5-8 min, linear gradient 5–50% (A); 8–12 min, linear gradient 50–90% (A); 12–14 min, linear gradient 90–100% (A); 14–16 min, isocratic gradient 100% (A). The flow rate was set at 0.4 mL/min with injection volume of 5 μL. The column temperature was 25°C. The effluent was further analyzed by Xevo G2-XS^®^ Quadrupole Time-of-Flight (Q-TOF) Mass Spectrometer with an ESI ion source in positive mode. The acquisition range was 50–1200 Da with 0.1 s scan rate. These analytic settings were aiming to reveal most of the chemicals within the herbal extracts.

### Cell Culture

RAW264.7 cell, a mouse blood macrophage cell line, was obtained from American Type Culture Collection (ATCC, Manassas, VA, United States). Cells were cultured in Dulbecco’s modified Eagles medium supplemented with 100 U/mL penicillin, 100 μg/mL of streptomycin and 10% heat in-active fetal bovine serum in a humidified CO_2_ (5%) incubator at 37°C. When the cells reached 80% confluence, they were harvested from plate with a scraper (Corning Incorporated, Corning, NY, United States).

### Cell Viability

Cell viability was measured by MTT assay. Cells were seeded in 96-well plates at a density of 1 × 10^4^ cells per well. After 24 h drug treatment, cells in each well were incubated with 10 μL MTT (5 mg/mL) at a final concentration of 0.5 mg/mL for 2 h at 37°C. After the solution was removed, DMSO was used to re-suspend the purple precipitate inside the cells, and the absorbance was detected at 570 nm. The cell viability was calculated as percentage of absorbance value of control (without drug treatment) while the value of control was 100%.

### Folin-Ciocalteu Assay

Total phenolic content of herbal extracts was measured with Folin-Ciocalteu assay. To be specific, 20 μL of each extract together with 40 μL 10% (*v*/*v*) Folin-Ciocalteu reagent was added into each well of 96-well microplate. Then, 160 μL Na_2_CO_3_ (700 mM) was added into each well. The assay plates were incubated at room temperature in dark for 2 h and then the absorbance at 765 nm were recorded. Here, gallic acid (Sigma-Aldrich, > 98%) was used as the reference compound, and the total phenolic contents of each extract were expressed as the value compared with gallic acid.

### DPPH Radical Scavenging Assay

Free radical scavenging activity of herbal extracts was measured with DPPH radical scavenging assay. Briefly, 50 μL of each extract with different concentrations (0–8 mg/mL) was mixed with 150 μL DPPH solution in each well of 96-well microplate. After standing for 10 min, the absorbance at 517 nm was recorded. The DPPH free radical scavenging activity was calculated as an inhibition percentage based on the following equation: Inhibition (%) = [(A_0_ - A_1_)/A_0_] × 100, where A_0_ is the absorbance of the control, and A_1_ is the absorbance of the RA sample aliquot. Here, gallic acid (0–100 μM) was used as a positive control.

### tBHP-Induced Oxidative Stress Assay

The dose of tBHP (150 μM; Sigma-Aldrich) and positive control (vitamin C, 1 mM) were optimized with MTT assay as previously reported (Huang et al., 2018a,b). Similar to the cell viability assay, the cells were cultured in 96-well plate first. After drug treatments for 24 h, tBHP (150 μM) were added into the wells for 3 h before MTT at a final concentration of 0.5 mg/mL was added. After the solution was removed, the purple precipitate inside the cells was re-suspended in DMSO and then measured at 570 nm absorbance.

### ROS Formation Assay

The measurement of ROS content in cell cultures was performed by using DCFH-DA, an oxidation-sensitive dye. Cultured RAW264.7 cells (1 × 10^4^ cells/well) in a 96-well plate were pre-treated with herbal extracts or standard compounds for 24 h, and the cells were labeled with 100 μM DCFH-DA (Sigma-Aldrich) in HBSS (Sigma-Aldrich) for 1 h at 37°C. After washing three times with HBSS, the cells were then treated with 150 μM tBHP for 1 h at 37°C. Then the amount of intracellular tBHP-induced ROS formation was detected by fluorometric measurement with excitation at 485 nm and emission at 530 nm.

### Assay for Anti-oxidant Response Element

To reveal the transcriptional activation of anti-oxidant response element (ARE), the pARE-Luc DNA construct, containing four copies of ARE (5′-TGACnnnGCA-3′) that drives transcription of the luciferase reporter gene luc2P (*Photinus pyralis*), was transfected into cultured RAW264.7 cells by Lipofectamine 3000 (Invitrogen) according to the manufacturer’s instructions. Transfected RAW264.7 cells were treated with various concentrations of ginseng extracts for 1 day. Then, the medium was aspirated, and the cultures were lysed by a buffer containing 100 mM potassium phosphate buffer (pH 7.8), 0.2% Triton X-100 and 1 mM DTT at 4°C. After centrifugation at 16,100 *× g* for 5 min, the supernatant was collected and used to perform luciferase assay. The activity was normalized as absorbance (up to 560 nm) per mg of protein.

### Statistical Analysis

HPLC profile processing were conducted using Agilent MassHunter workstation software version B.01.00. The ^1^H NMR spectra obtained from each sample were phased, baseline-corrected, calibrated and integrated using MestReNova 6.1.1 software. LC-MS profile were analyzed with Waters MassLynx V4.1 SCN923. The data were then formatted in XML for importing into PCA software SIMCA-P+ version 12.0 (Umetrics, Sweden). All data were expressed as the mean ± SEM for *n* = 3–5, unless otherwise specified. Statistical tests were performed by one-way ANOVA with multiple comparisons using Dunnett’s test. Differences were considered significant at *p* < 0.05.

## Results

### Chemical Composition of Herbal Extracts

**FIGURE 1:**
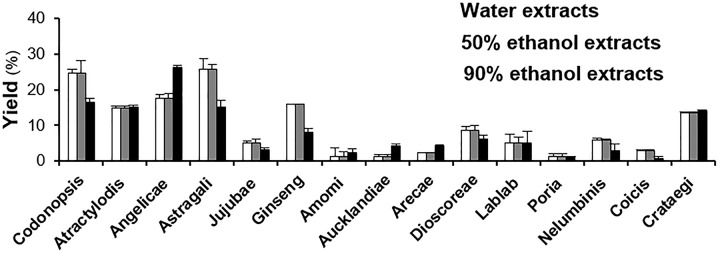
The yields of water extract, 50% ethanol extract and 90% ethanol extract of 15 spleen-meridian herbs. The herbal extracts were prepared with distilled water, 50% ethanol and 90% ethanol by refluxing twice and each time for 2 h. The yields were calculated as the percentage of the weight of dried extracts to herbal powders. All values are in Mean ± SD, *n* = 3. The detailed yield and origin of each batch were shown in Table 1.

By reflux, the water extracts and ethanol extracts of individual herbs were prepared with the consideration of their common usage and historical preparation of a herbal decoction in TCM. The origin of herbs and its extracting yields are shown in Table 1. The extracting yield of Codonopsis Radix, Astragali Radix, Jujubae Fructus, Ginseng Radix, Nelumbinis Semen and Coicis Semen suggested that the water-soluble ingredients in these herbs were relatively abundant; while Angelicae Sinensis Radix, Amomi Fructus, Aucklandiae Radix and Areca Catechu contained less water-soluble ingredients (Figure 1). Among the tested herbs, Codonopsis Radix, Angelicae Sinensis Radix and Astragali Radix possessed the highest yield of extraction up to ∼25% per dried weight. In contrast, the yields of water and ethanol extracts of Amomi Fructus, Poria Cocos and Coicis Semen were less than 3%.

**FIGURE 2:**
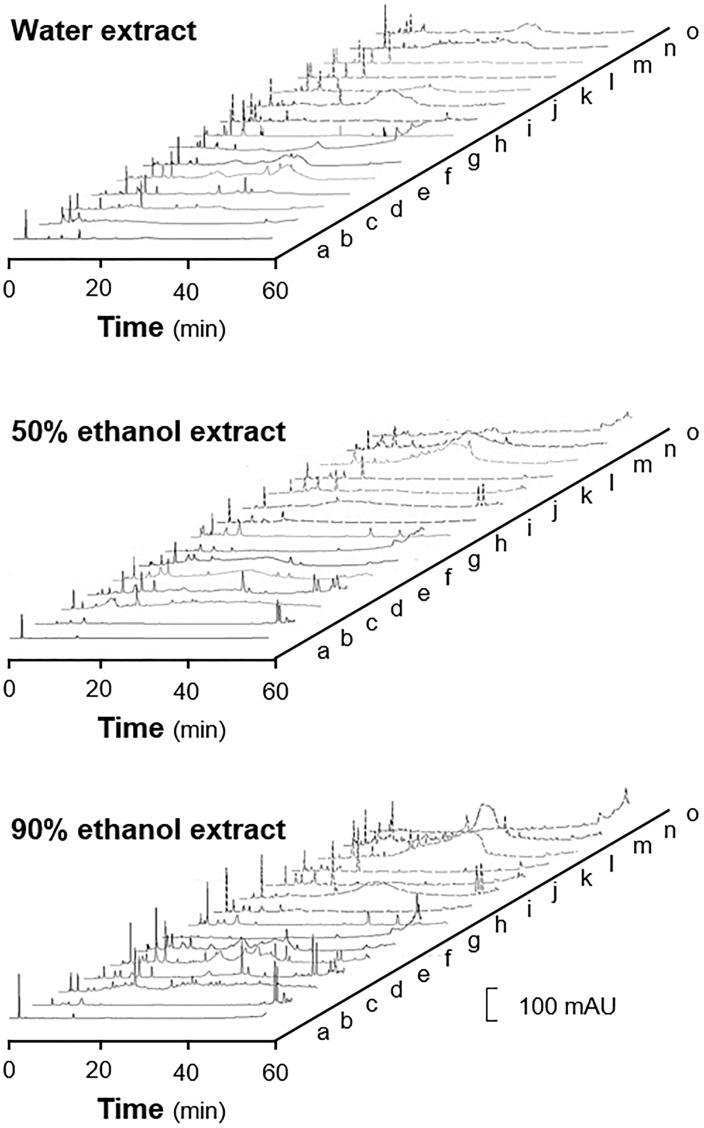
HPLC profiles of herbal extracts from the 15 collected herbs. The fingerprints were obtained with an Agilent HPLC 1200 series system (Agilent, Waldbronn, Germany) and a Waters Symmetry C18 column (4.5 mm × 250 mm, 5 μm i.d.). The mobile phase was composed of acetonitrile and water, and the wavelength of UV detector was set to 254 nm with full spectral scanning from 190 to 400 nm. The extracting solvents are indicated. The extracts from 3 batches of herbs were subjected to analysis, a representative profile was shown. a, Codonopsis Radix; b, Atractylodis Macrocephalae Rhizoma; c, Angelicae Sinensis Radix; d, Astragali Radix; e, Jujubae Fructus; f, Ginseng Radix; g, Amomi Fructus; h, Aucklandiae Radix; i, Areca Catechu; j, Dioscoreae Ahizoma; k, Lablab Semen Album; l, Poria Cocos; m, Nelumbinis Semen; n, Coicis Semen; o, Crataegi Fructus.

**FIGURE 3:**
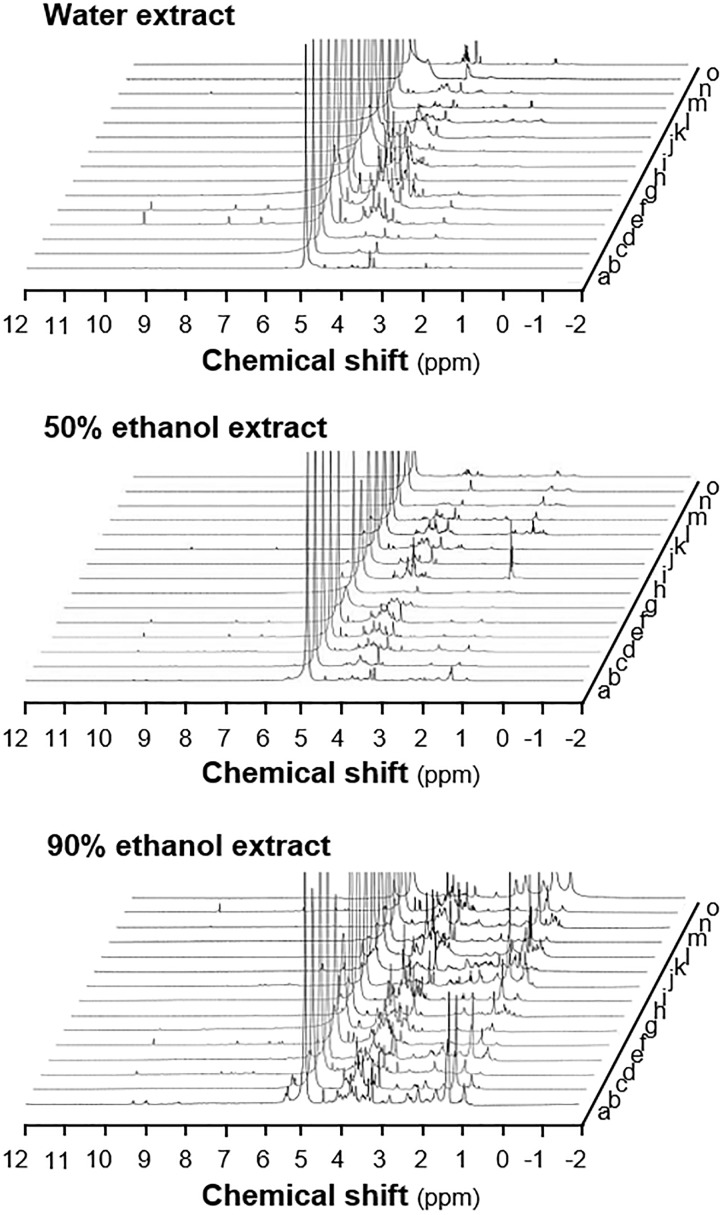
^1^H NMR profiles of herbal extracts from the 15 spleen-meridian herbs. The extracts (50 mg) were dissolved in 400 μL of D_2_O and centrifuged at 13,000 *× g* for 1 min before transferred to a standard 5-mm NMR tube. The ^1^H NMR experiment were conducted on a Varian 300 MHz NMR spectrometer, operating at 300.13 MHz ^1^H NMR frequency at 298 K. The spectra were acquired using a 1D CPMG pulse sequence (RD-90°-t1-90°-tm- 90°-acquire) to generate a spectrum with a reduced residual solvent peak. The range of chemical shift was set from –2 to 12 ppm, and the deuterated solvent peak at 4.8 was set as calibration. The extracting solvents are indicated. The extracts from 3 batches of herbs were subjected to analysis, a representative profile was shown. a, Codonopsis Radix; b, Atractylodis Macrocephalae Rhizoma; c, Angelicae Sinensis Radix; d, Astragali Radix; e, Jujubae Fructus; f, Ginseng Radix; g, Amomi Fructus; h, Aucklandiae Radix; i, Areca Catechu; j, Dioscoreae Ahizoma; k, Lablab Semen Album; l, Poria Cocos; m, Nelumbinis Semen; n, Coicis Semen; o, Crataegi Fructus.

The HPLC fingerprints of water and ethanol extracts showed that there were plenty of trace compounds (Figure 2). By comparing the HPLC profiles, the 90% ethanol extracts in general contained higher amounts of trace compounds, recognized by the number of peaks, as compared to that of water and 50% ethanol extracts. The distribution of peaks in HPLC profiles showed apparent migration in accord to decreasing polarity of extracting solvent, i.e., more peaks at the end of run in ethanol extracts (Figure 2). The ^1^H NMR experiment was conducted to reveal those high abundance compounds in aforementioned herbal extracts. Similar to HPLC fingerprint, the NMR profile showed close similarity between herbs extracted with the same solvents, and generally the 90% ethanol extracts showed higher content of chemical composition, as compared with that of other extracts (Figure 3).

**FIGURE 4:**
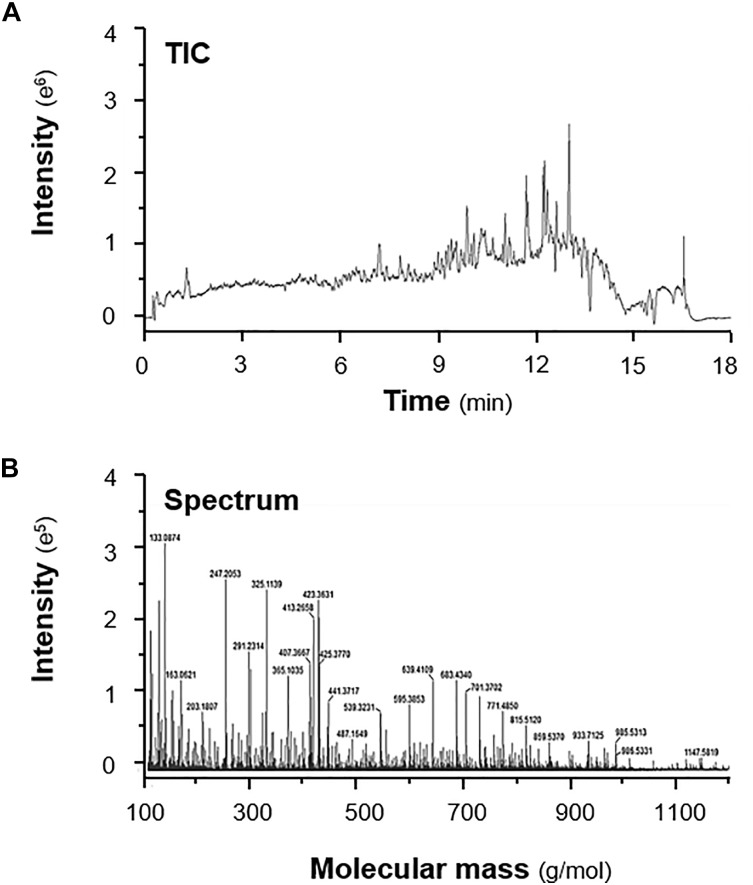
A typical total ion chromatogram (TIC) and mass spectrum. The 90% ethanol extract of Ginseng was served as an example. **(A)** Total ion chromatogram (TIC) and **(B)** mass spectrum of the Ginseng extracts are shown. The X-axis of TIC is time (min); while the X-axis of spectrum is mass (Da). Both profiles were obtained with a Xevo G2-XS^®^ Quadrupole Time-of-Flight (Q-TOF) Mass Spectrometer coupled with an integrated ACQUITY UPLC^®^ I-Class System. The mobile phase was composed of acetonitrile and water, and the effluent was analyzed with an ESI ion source in positive mode. The acquisition range was set from 50 to 1200 Da with 0.1 s scan rate to reveal most of the chemicals within the herbal extracts. The extracts from 3 batches of herbs were subjected to analysis, a representative profile was shown.

High resolution mass spectrometry was established in analyzing the herbal extracts. The LC-MS profiles could be displayed in two forms: total ion chromatogram (TIC) and mass spectrum. Figure 4 shows a typical profile of 90% ethanol extract of Ginseng Radix. The distribution of trace ingredients with different polarity and molecular mass were shown. A list of mass spectrum with intensity was obtained for the herbal extracts. The LC-MS profiles of 15 spleen-meridian herbs were established, and the data processing was performed on Waters MassLynx V4.1 SCN923. With MassLynx, each chromatographic peak was identified by a mass-to-charge ratio (*m*/*z*) and retention time, as illustrated in Supplementary Figure 1. As shown in Table 2, 65 compounds in 15 herbs were identified with unique relative molecular mass of [M + H]^+^, [M + Na]^+^ or [2M + H]^+^ within acceptable experimental error, which therefore could be used for the purpose of chemical authentication.

**Table 2:** The compounds identified from the herbal extracts.

Herbs	Retention time (t_R_, min)	Identification	[M + H]^+^ (*m*/*z*)	Molecular weight (Da)	Formula	Mass error (ppm)
Codonopsis Radix	0.942	Codonopsine	268.1614	267.1549	C_14_H_21_NO_4_	5.4
	2.323	Vanillic acid	169.0581	168.0494	C_8_H_8_O_4_	4.5
	3.565	Tryptophan sulfate	286.0615	285.054	C_11_H_12_N_2_O_5_S	1.5
	5.932	Ferulic acid	196.0743	195.0665	C_10_H_10_O_4_	0.7
	9.921	Atractylenolide II	234.1618	233.1538	C_15_H_20_O_2_	0.3
	9.936	Codonopyrrolidium A	351.4372	350.429	C_19_H_28_NO_5_	0.7
Atractylodis	5.903	Icariside D1	417.1715	416.1687	C_19_H_28_O_10_	12.4
Macrocephalae	7.382	Atractylenolide III	271.1321^a^	248.1417	C_15_H_20_O_3_	2.2
Rhizoma	7.749	Atractylenolactam	230.1557	229.1471	C_15_H_19_ON	2.9
	8.087	Atractyloside A	449.2399	448.2313	C_21_H_36_O_10_	1.5
	8.145	Atractyloside B	473.2363^a^	450.246	C_21_H_38_O_10_	1.1
	8.796	*β*-sitosterol	415.3944	414.3867	C_29_H_50_O	0.6
	11.231	Atractylenolide I	233.1544	232.1467	C_15_H_20_O_2_	1
	12.26	Atractylenolide II	231.1381	230.1311	C_15_H_18_O_2_	4.1
	12.26	Atractylenolide VI	203.1807	202.1726	C_15_H_22_	0.8
Angelicae Sinensis Radix	9.583	Ferulic acid	196.0759	195.0659	C_10_H_10_O_4_	10.6
	10.797	Levistolide A	382.2154	381.2066	C_24_H_28_O_4_	2.3
	12.256	Linoleic acid	280.2395	279.2314	C_18_H_32_O_2_	0.6
Astragali Radix	4.453	Calycosin	285.2673	284.2634	C_16_H_12_O_5_	14.2
	4.608	Wogonin	285.2673	284.2636	C_16_H_12_O_5_	14.9
	5.987	Formononetin	269.2712	268.264	C_16_H_12_O_4_	2.8
	6.001	Astragaloside II	828.0103	827.0069	C_43_H_70_O_15_	5.5
	6.339	Astragaloside IV	785.9793	784.9702	C_41_H_68_O_14_	1.5
	7.03	Soyasaponin I	944.1336	943.1221	C_48_H_78_O_18_	3.8
	9.821	Isorhamnetin	317.2684	316.2623	C_16_H_12_O_7_	5.8
	10.274	Astragaloside VII	948.1183	947.1108	C_47_H_78_O_19_	0.5
	10.275	Soyasaponin II	914.1056	913.0961	C_47_H_76_O_17_	1.7
	11.724	Medicarpin	271.2863	270.28	C_16_H_14_O_4_	6.1
Jujubae Fructus	2.154	Coclaurine	286.1543	285.1443	C_17_H_19_NO_3_	7.2
	2.334	Magnoflorine	342.1794	341.1705	C_20_H_24_NO_4_	2.8
	4.154	Juzirine	282.1187	281.113	C_17_H_15_NO_3_	8
	4.636	Spinosin	609.1878	608.1819	C_28_H_33_O_15_	3.4
Ginseng Radix	2.705	Ginsenoside Rg_2_	785.5016	784.4973	C_42_H_72_O_13_	4.6
	5.27	Ginsenoside Rh_2_	623.4571	622.4445	C_36_H_62_O_8_	7.5
Amomi Fructus	3.126	Isoquercitrin	465.3867	464.38	C_21_H_20_O_12_	2.7
	3.508	Quercetin	449.3878	448.3769	C_21_H_20_O_11_	6.6
Aucklandiae Radix	6.439	*β*-caryophyllene	205.3671	204.36	C_15_H_24_	4.1
	9.936	Alantolactone	233.3176	232.31	C_15_H_20_O_2_	1.5
Areca Catechu	8.087	Catechin	291.2758	290.27	C_15_H_14_O_6_	7.4
	13.009	Epigallocatechin	307.2769	306.27	C_15_H_14_O_7_	3.4
Dioscoreae Ahizoma	13.135	Pseudoprotodioscin	1031.507	1030.5	C_51_H_82_O_21_	3.4
Lablab Semen Album	9.77	Arachidic acid	313.3159	312.3028	C_20_H_40_O_2_	3.4
Poria Cocos	6.666	Dehydrotumulosic acid	485.3644	484.3553	C_31_H_48_O_4_	2.4
	7.087	Mechylcis-9-hexadecenoate	269.4418	268.4348	C_17_H_32_O_2_	3.5
	9.184	Hederagenin	473.3619	472.3553	C_30_H_48_O_4_	2.8
	9.202	Pachymic acid	529.3865	528.3815	C_33_H_52_O_5_	5.6
	10.994	Dehydropachymic acid	527.3738	526.3658	C_33_H_50_O_6_	0.1
Nelumbinis Semen	10.36	Naringenin	273.0734	272.0685	C_15_H_12_O_5_	11.2
Coicis Semen	2.618	1,3-diolein	621.9989	620.986	C_39_H_72_O_5_	8
	13.502	Trilinolein	880.3988	879.384	C_57_H_98_O_6_	7.8
	14.544	Triolein	886.4386	885.432	C_57_H_104_O_6_	1.5
Crataegi Fructus	2.661	Quercetin 3-*O*-(6-*O*-rhamnosyl-glucoside) (rutin)	611.1526	610.1458	C_27_H_30_O_16_	1.9
	2.677	Quercetin 3-*O*-(2, 6-di-*O*-rhamnosyl-glucoside)	757.2094	756.2037	C_33_H_40_O_20_	3
	2.846	(epi)afzelechin-(epi)catechin	563.1459	562.1402	C_30_H_26_O_11_	4
	3.097	Quercetin	303.0414	302.0349	C_15_H_10_O_7_	4.8
	4.636	Diosmetin 7-*O*-rutinoside (diosmin)	609.1737	608.1665	C_28_H_32_O_15_	1.2
	4.64	Quercetin 3-*O*-rhamnoside (quercitrin)	448.0975	447.0928	C_21_H_20_O_11_	7.2
	9.3	Pectolinarin	623.1901	622.1827	C_29_H_34_O_15_	0.9
	9.767	Apigenin 7-*O*-glucoside	433.1032	432.0979	C_21_H_20_O_10_	6.1
	10.314	Quinic acid	193.0655	192.0561	C_7_H_12_O_6_	7.6
	10.343	Epicatechin	291.0789	290.0716	C_15_H_14_O_6_	2.2
	10.512	Isorhamnetin-*O*-hexoside	479.1131	478.1036	C_22_H_22_O_12_	3.3
	10.994	Phloretin 2′-*O*-glucoside (phlorizin)	437.1365	436.1294	C_21_H_24_O_10_	1.9
	11.724	3-*O*-caffeoylshikimic acid	337.0856	336.0773	C_16_H_16_O_8_	1.1
	12.361	*p*-coumaric acid-*O*-hexoside	327.1006	326.0927	C_15_H_18_O_8_	0.1


*^a^Mass-to-charge ratio (*m*/*z*) is [M + Na]^+^.*

### Principal Component Analysis of Chemical Composition Profiles

**FIGURE 5:**
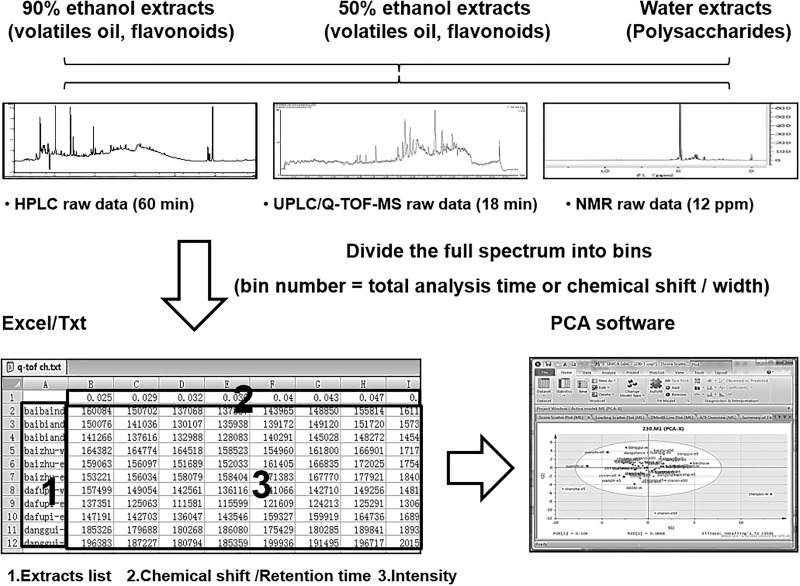
The procedure of full spectrum principle component analysis. The spectrums were firstly divided into bins according to the resolution of analytic methods with its corresponding softwares (Agilent MassHunter workstation software version B.01.00 for HPLC, Waters MassLynx V4.1 SCN923 for LC-MS and MestReNova 6.1.1 for NMR). The intensity of each bin was extracted and arranged as the figure showed. The obtained excel or txt file was further subjected into PCA software (SIMCA-P+ version 12.0, Umetrics, Sweden).

To determine the similarities and differences among the herbal extracts, PCA analyses of chemical profiles deriving from HPLC, NMR and LC-MS were conducted. PCA is a chemometrics method to determine the possible relationship of different samples. The general procedure of PCA on full spectrum was shown in Figure 5. The spectrums were firstly divided into bins according to the resolution of analytic methods, i.e., 9,000 bins for HPLC, 1,200 bins for NMR, 10,800 bins for TIC and 230,000 bins for mass spectrum, with analytic softwares, e.g., Agilent MassHunter workstation software version B.01.00 for HPLC, Waters MassLynx V4.1 SCN923 for LC-MS and MestReNova 6.1.1 for NMR. The intensity of each bin was extracted and arranged. The obtained excel or txt files were further imported into PCA software (SIMCA-P+ version 14.1) for chemometrics analysis.

**FIGURE 6:**
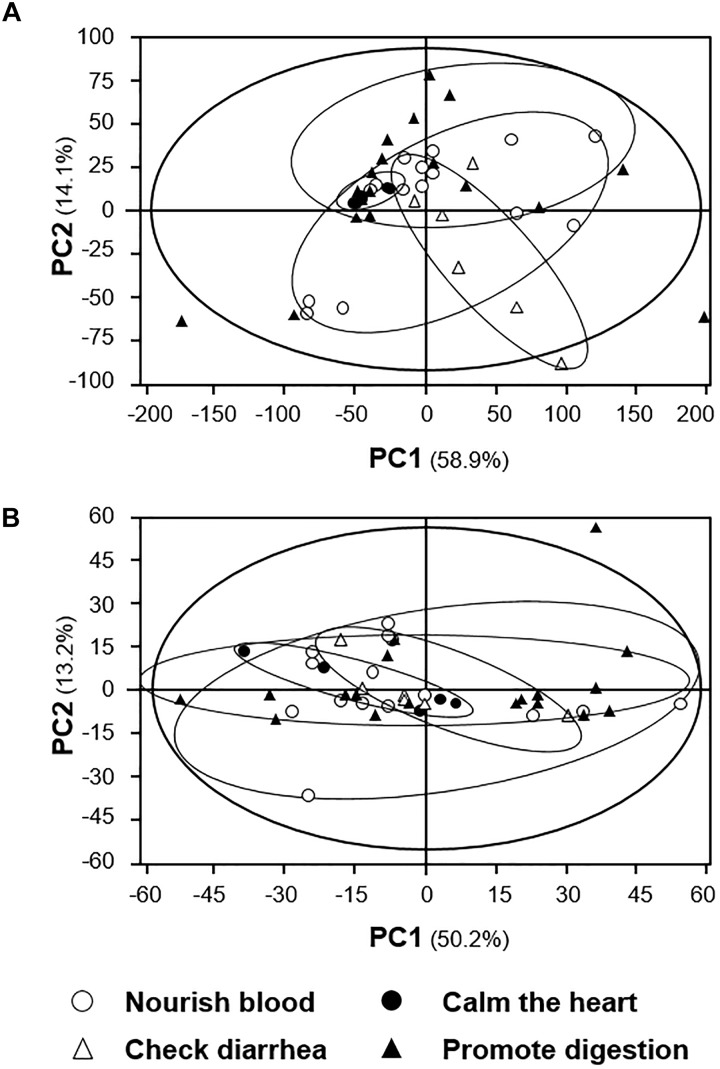
PCA analyses of **(A)** HPLC and **(B)** ^1^H NMR profiles of signals from the 15 spleen-meridian herbs. The distribution of each herbal extract in score scatter plots was calculated according to its correlation with the major components. The classification of herbal extracts was according to the proposed pharmacological activity, as described in TCM practice. The results showed very low discrimination between different types of herbal extracts.

According to the TCM theory, the selected 15 spleen-meridian herbs could not only be divided into three different groups according to their “Yin-Yang” nature, and which also could be divided into four groups according to their pharmacological activities (Table 1). In clinical application, these herbs nourish the blood, calm the heart and promote the digestion. By characterizing the relationship between the distributions of herbal extracts in score scatter plots with their identified pharmacological activities, the correlation between chemical composition basis and known pharmacology could be clarified. Although the score scatter plots of HPLC fingerprints (Figure 6A) and NMR profiles (Figure 6B) possessed acceptable first principal component (58.9%, 50.2%) and second principal component (14.1%, 13.2%), the distribution of herbal extracts showed no apparent regular pattern among different groups. The results indicated that HPLC and NMR profiles might not able to distinguish the herbal extracts with different pharmacological properties.

**FIGURE 7:**
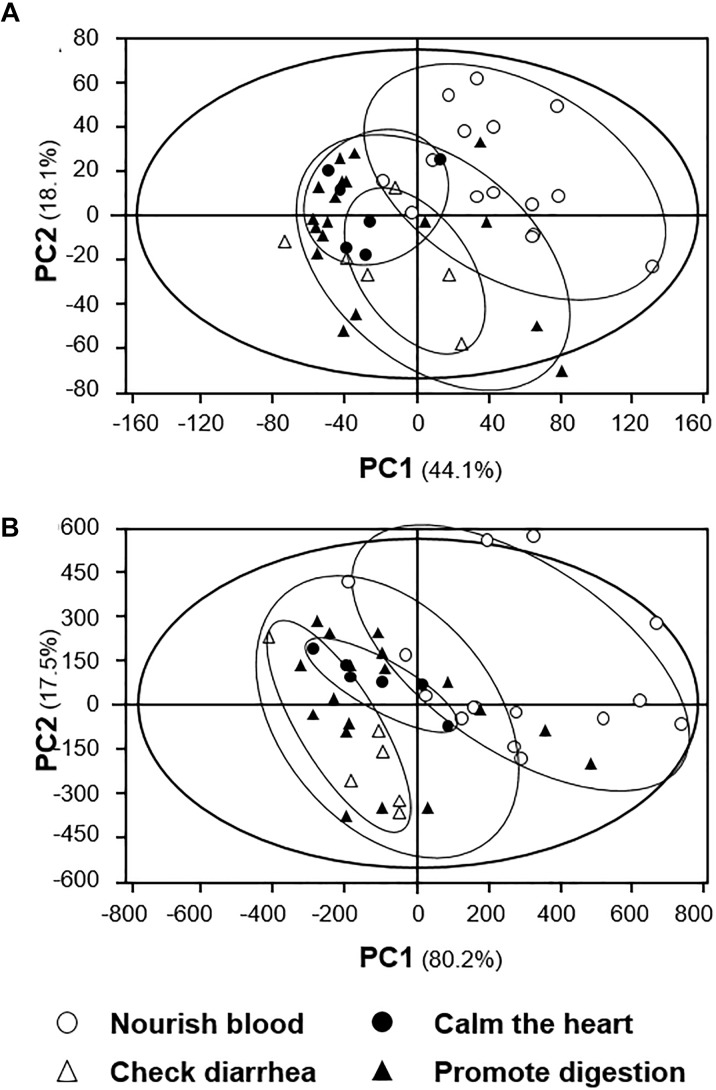
PCA analyses of **(A)** TIC and **(B)** spectrum profiles of signals from the 15 spleen-meridian herbs. Similarly, the distribution of each herbal extract in score scatter plots was calculated according to its correlation with the major components. The classification of the herbal extracts was according to the proposed pharmacological activity, as described in TCM practice. The results showed relatively higher discrimination between different types of extracts.

The LC-MS profiles, including TIC and mass spectrum obtained from UPLC/Q-TOF, were further analyzed with the same procedures as that of HPLC and NMR profiles. Both score scatter plots possessed acceptable first principal component and second principal component, especially in the scenario of two principal components of mass spectrum accounting for 97.7% of total variance (Figures 7A,B). These results therefore could be an outcome of ultra-high resolution of UPLC/Q-TOF. The two PCA scoring plots showed similar distribution according to their identified pharmacological activities, indicating that the polarity of herbal compounds might have positive correlation with their mass, at least to certain degree. Moreover, the herbal extracts having similar pharmacological activities showed a clustering effect. In the clustering analysis, the blood nourishing herbs could be distinguished obviously from other herbs (Figure 7). Thus, the plots showed a strong correlation between distribution of herbal extracts with their pharmacological activities, which was in line with the hypothesis that the bioactivities of herbal extracts should be strongly related to their chemical composition.

**FIGURE 8:**
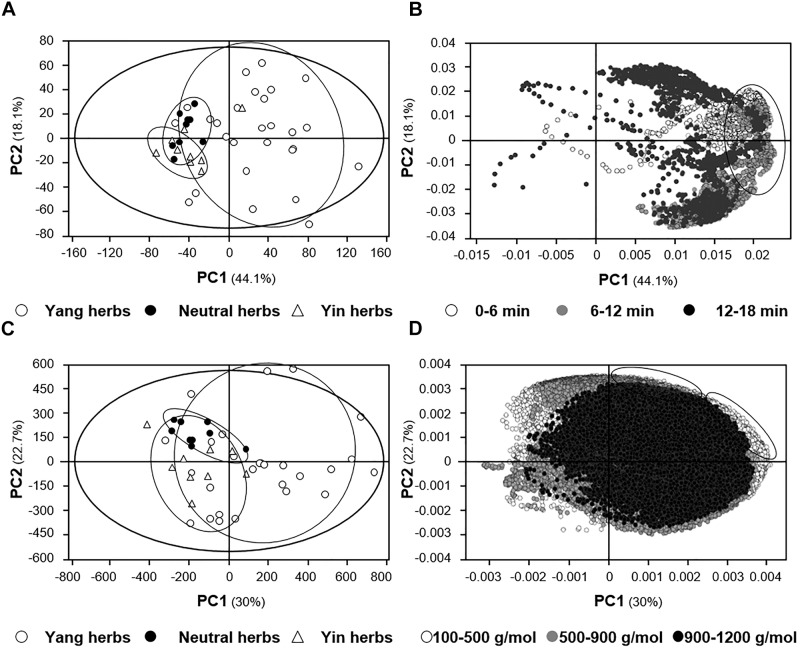
PCA analyses of LC-MS profiles of signals from the 15 spleen-meridian herbs. **(A)** Scoring plots of TIC. **(B)** Loading scatter plots of TIC. **(C)** Scoring plots of spectrum. **(D)** Loading scatter plots of spectrum. The distribution of each herbal extract in score scatter plots was calculated according to its correlation with the major components. The classification of herbal extracts was according to the “Yin-Yang” nature of herbs, i.e., Yin, neutral and Yang herbs, according to TCM practice. The results showed very obvious discrimination between herbal extracts with different properties. Each dot in the loading scatter plots represents a variable. Dots in **(B)** represent time intervals for 0.1 s, and dots in **(D)** represent mass intervals of 0.005 Da.

The relationship between “Yin-Yang” nature and distribution of the herbal extracts in scoring plots were determined. The distribution of Yin, Yang and neutral herbs were significantly identified to be different, in particular the right part of score scatter plots contained only the Yang-stimulating herbs (Figures 8A,C). To clarify the chemical basis of distinct distribution, the variables in loading scatter plots could be divided into three clustering groups, according to their retention time and molecular mass. From the distribution of herbal extracts in score scatter plot, the contents of compounds eluted between 6 and 12 min showed greater impacts in the “Yin-Yang” distinction of herbal extracts (Figure 8B). Similarly, the lighter molecules, i.e., less than 900 g/mol, were distributed in marginal positions in the loading scatter plots (Figure 8D), indicating that these compounds might have obvious influence to the herbs’ properties. Moreover, it could be deduced that the Yang-stimulating herbs were shown to contain more compounds, i.e., the compounds with higher polarity and lower molecular mass. These results not only illustrated the chemical basis in classify “Yin-Yang” nature of TCM, and which also suggested the polarity and molecular mass of ingredients in the herbs could be two major influencing factors.

**FIGURE 9:**
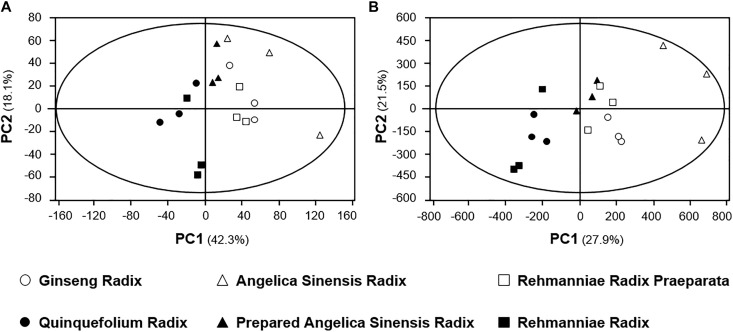
PCA analyses of **(A)** TIC and **(B)** spectrum profiles of three paired-herbs with typical cold or hot property, i.e., Ginseng Radix-Quinquefolium Radix, Angelica Sinensis Radix-Prepared Angelica Sinensis Radix, Rehmanniae Radix Praeparata-Dried Rehmanniae Radix. The distribution of each herbal extract in score scatter plots was calculated according to its correlation with the major components.

To determine applicability of present analytic methods, three pair-herbs having typical cold and hot properties, as recorded in TCM practice, were subjected into our developed PCA analysis. Ginseng Radix and Rehmanniae Radix Praeparata are considered as hot and warm herbs aiming to tonify qi and to nourish blood; while Quinquefolium Radix and Rehmanniae Radix are cold herbs used to clear heat aiming to promote fluid production (Yu et al., 2001; Zhang et al., 2008). Angelica Sinensis Radix is a typical herb in invigorating blood in TCM therapy. However, the treatment of Angelica Sinensis Radix with wine reduces the amount of volatile oil, and this treatment could directly minimize the hot property of Angelica Sinensis Radix (Zhan et al., 2011). These three paired-herbs have the same or similar origin, and however, they have opposite functions. From the PCA score scatter plots of TIC and spectrum, the distribution of herbal extracts with hot or warm properties (i.e., Ginseng Radix and Rehmanniae Radix Praeparata) were significantly different from the herbs having cold or cool properties (i.e., Quinquefolium Radix and Rehmanniae Radix) (Figures 9A,B). As expected, the polarity and molecular mass of major components in the extracts of pair-herbs were different, accordingly. Although the difference between Angelica Sinensis Radix and wine-treated Angelica Sinensis Radix in pharmacological properties was not as obvious as other paired-herbs, as mentioned above, the distribution of the two herbal extracts could also be distinguished significantly in the score scatter plots (Figures 9A,B). The result strongly supported the precision applicability of the present established analytical model.

### Anti-oxidative Profile of Spleen-Meridian Herbs

**FIGURE 10:**
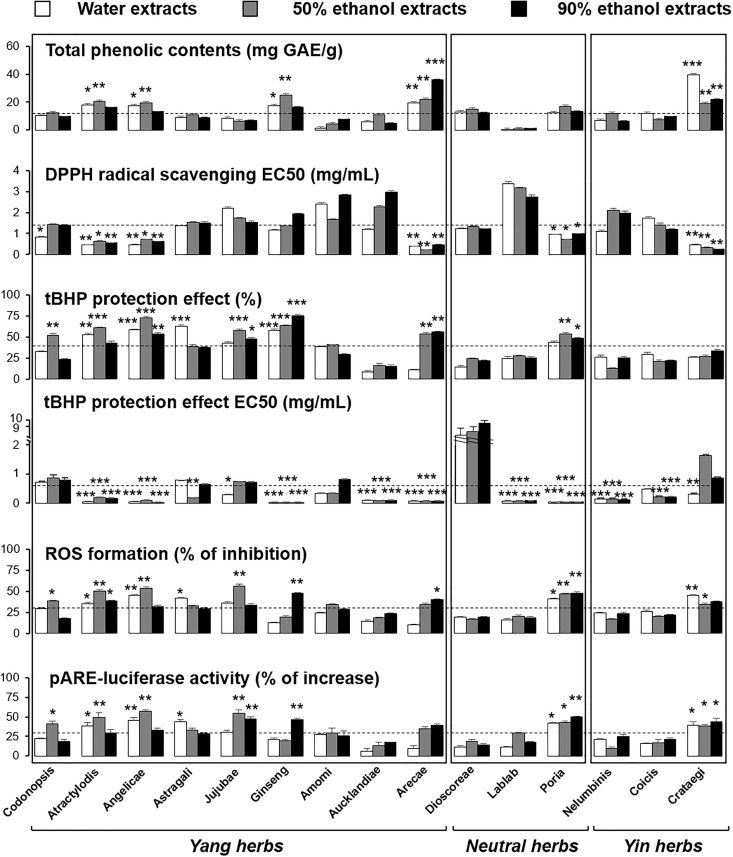
The correlation between anti-oxidative profiles of the spleen-meridian herbs with the “Yin-Yang” classification criterion. Values are expressed as Mean ± SD, where *n* = 4, each with triplicate samples. Statistical comparison was made with mean value. The mean value of total phenolic compounds was 12.87 mg GAE/g; mean value of DPPH radical scavenging EC50 was 1.38 mg/mL; mean value of tBHP protection effect was 37.9%; mean value of tBHP protection effect at IC50 was 0.65 mg/mL; mean value of inhibition effects to ROS formation was 30.5%; mean value of pARE-luciferase activity increase was 29.6%. ^∗^*p* < 0.05; ^∗∗^*p* < 0.01; ^∗∗∗^*p* < 0.001.

To elucidate the connection between the “Yin-Yang” nature and redox system, a series of *in vitro* assays were conducted to measure the anti-oxidative properties of herbal extracts. Folin-Ciocalteu assay was used to determine total phenolic compounds in herbal extracts. Phenolic compounds, highly abundant in plant, generally have unstable electrons in the phenolic hydroxyl groups, and which protect cells from being oxidized by directly neutralizing free radicals or decomposing peroxides. The content of total phenolic compounds in the selected herbs were rather similar (Figure 10 and Table 3). In exception, the water and 50% ethanol extracts of Lablab Semen Album contained much less of total phenolic content (Figure 10). In contrast, Arecae Pericarpium and Crataegi Fructus possessed relative higher contents, as compared with other herbs. The 50% ethanol extracts of herbs contained higher amounts of phenolic compounds in general, except in the scenario of Aucklandiae Radix and Nelumbinis Semen (Figure 10).

**Table 3:** The correlation between anti-oxidant profiles of the spleen-meridian herbs with the “Yin-Yang” classification criterion.

AttributesHerbs		Total phenolics (mg GAE/g)^b^			DPPH radical scavenging EC50 (mg/mL)			tBHP protection(%)			tBHP protection EC50 (mg/mL)			ROS formation (% of inhibition)			pARE-luciferase activity (% of increase)		
						
		W^c^	E50	E90	E90	E50	E90	W	E50	E90	W	E50	E90	W	E50	E90	W	E50	E90
Yang herbs	CR^a^	10.21 ± 0.32^d^	12.25 ± 0.22	9.71 ± 0.21	0.82 ± 0.03^∗^	1.44 ± 0.04	1.39 ± 0.05	32.7 ± 0.97	51.8 ± 2.34^∗∗^	23.6 ± 0.92	0.72 ± 0.05	0.88 ± 0.1	0.8 ± 0.1	29.72 ± 1.22	38.37 ± 1.41^∗^	17.41 ± 1.18	21.82 ± 1.72	40.94 ± 4.09^∗^	18.21 ± 2.82
	AMR	17.91 ± 0.32^∗^	20.31 ± 0.24^∗∗^	16.19 ± 0.23	0.46 ± 0.01^∗∗^	0.64 ± 0.02^∗^	0.55 ± 0.02^∗∗^	53 ± 1.65^∗∗^	61.3 ± 0.67^∗∗∗^	42.8 ± 2.53	0.05 ± 0.01^∗∗∗^	0.2 ± 0^∗∗∗^	0.18 ± 0^∗∗∗^	35.28 ± 1.39^∗^	50.36 ± 1.89^∗∗^	38.14 ± 1.3^∗^	38.68 ± 4.19^∗^	49.04 ± 6.19^∗∗^	29.82 ± 3.8
	ASR	17.61 ± 0.22^∗^	19.5 ± 0.26^∗∗^	13.25 ± 0.11	0.47 ± 0.02^∗∗^	0.72 ± 0.02^∗^	0.62 ± 0.03^∗^	58.4 ± 1.21^∗∗∗^	73.1 ± 1.63^∗∗∗^	53.6 ± 1.71^∗∗^	0.06 ± 0.01^∗∗∗^	0.1 ± 0^∗∗∗^	0.03 ± 0.01^∗∗∗^	45.01 ± 1.46^∗∗^	53.61 ± 1.57^∗∗^	31.89 ± 1.33	45.75 ± 3.15^∗∗^	57.02 ± 2.57^∗∗^	32.62 ± 3.26
	AsR	9.02 ± 0.21	10.93 ± 0.31	8.92 ± 0.21	1.36 ± 0.02	1.52 ± 0.04	1.5 ± 0.06	62.5 ± 1.87^∗∗∗^	38.7 ± 2.39	37.6 ± 0.82	0.8 ± 0.01	0.19 ± 0^∗∗^	0.67 ± 0	41.72 ± 1.44^∗^	32.46 ± 1.33	29.63 ± 1.29	43.72 ± 2.37^∗^	33.48 ± 1.85	28.86 ± 1.89
	JF	8.45 ± 0.31	6.18 ± 0.11	7.02 ± 0.21	2.19 ± 0.08	1.75 ± 0.03	1.53 ± 0.06	42.5 ± 1.93	57.7 ± 1.92^∗∗∗^	47.8 ± 1.28^∗^	0.3 ± 0.01^∗^	0.75 ± 0	0.74 ± 0.01	36.12 ± 1.3	56.49 ± 1.75^∗∗^	33.37 ± 1.48	30.33 ± 3.03	54.54 ± 4.54^∗∗^	47.64 ± 2.48^∗∗^
	GR	17.21 ± 0.33^∗^	24.91 ± 0.43^∗∗^	16.23 ± 0.43	1.17 ± 0.03	1.36 ± 0.02	1.94 ± 0.03	57.7 ± 1.76^∗∗∗^	63.3 ± 1.45^∗∗∗^	75.6 ± 1.13^∗∗∗^	0.04 ± 0.01^∗∗∗^	0.04 ± 0.01^∗∗∗^	0.04 ± 0.01^∗∗∗^	12.47 ± 1.12	19.44 ± 1.2	47.46 ± 1.47^∗∗^	21.67 ± 1.22	19.82 ± 1.4	46.81 ± 1.57^∗∗^
	AF	1.11 ± 0.02	4.17 ± 0.03	7.89 ± 0.07	2.41 ± 0.05	1.66 ± 0.05	2.83 ± 0.06	38.4 ± 1.08	40.7 ± 0.57	29.6 ± 0.81	0.34 ± 0	0.36 ± 0.01	0.83 ± 0	24.16 ± 1.27	34.03 ± 1.29	28.13 ± 1.26	27.47 ± 0.87	29.38 ± 6.29	26.28 ± 5.55
	AuR	5.94 ± 0.23	11.06 ± 0.36	5.03 ± 0.27	1.19 ± 0.05	2.27 ± 0.07	2.98 ± 0.07	8.5 ± 1.45	16.3 ± 1.91	15.2 ± 1.91	0.1 ± 0^∗∗∗^	0.1 ± 0^∗∗∗^	0.1 ± 0^∗∗∗^	14.56 ± 1.14	18.55 ± 1.17	23.23 ± 1.22	5.63 ± 3.63	13.63 ± 3.98	17.4 ± 0.67
	AC	19.28 ± 0.32^∗∗^	21.73 ± 0.34^∗∗^	35.97 ± 0.46^∗∗∗^	0.39 ± 0.01^∗∗^	0.22 ± 0.01^∗∗^	0.48 ± 0.02^∗∗^	11.1 ± 0.27	53.4 ± 1.85^∗∗^	56.3 ± 0.36^∗∗^	0.09 ± 0^∗∗∗^	0.08 ± 0^∗∗∗^	0.08 ± 0^∗∗∗^	9.88 ± 1.1	34.71 ± 1.34	39.94 ± 1.39^∗^	9.59 ± 3.96	34.4 ± 3.44	39.17 ± 2
Neutral herbs	DA	12.79 ± 0.44	15.1 ± 0.54	12.45 ± 0.52	1.24 ± 0.01	1.34 ± 0.02	1.23 ± 0.01	14 ± 1.65	24.2 ± 0.51	21.4 ± 1.44	4.43 ± 0.07	5.04 ± 0.06	6.5 ± 0.06	19.26 ± 1.12	16.84 ± 1.19	19.06 ± 1.14	11.74 ± 1.12	19.06 ± 1.91	14.13 ± 1.41
	LSA	0.14 ± 0.01	0.72 ± 0.02	1.14 ± 0.03	3.39 ± 0.09	3.17 ± 0.05	2.74 ± 0.09	24.4 ± 2.05	27.5 ± 0.92	25 ± 1.94	0.09 ± 0^∗∗∗^	0.09 ± 0^∗∗∗^	0.09 ± 0^∗∗∗^	16.32 ± 1.11	20.56 ± 1.3	18.65 ± 1.18	11.42 ± 1.01	29.54 ± 0.9	17.73 ± 0.77
	PC	12.57 ± 0.54	16.84 ± 0.51	13.31 ± 0.41	0.95 ± 0.01^∗^	0.74 ± 0.01^∗^	0.99 ± 0.01^∗^	43.7 ± 1.52	53.4 ± 2.2^∗∗^	48.6 ± 0.85^∗^	0.04 ± 0.01^∗∗∗^	0.04 ± 0.01^∗∗∗^	0.04 ± 0^∗∗∗^	40.81 ± 1.42^∗^	46.67 ± 1.43^∗∗^	47.86 ± 1.5^∗∗^	41.88 ± 0.82^∗^	43.03 ± 1.93^∗^	49.92 ± 0.8^∗∗^
Yin herbs	NS	6.97 ± 0.32	12.09 ± 0.34	6.58 ± 0.42	1.11 ± 0.06	2.12 ± 0.09	1.98 ± 0.08	26.2 ± 1.92	12.6 ± 0.71	25.2 ± 1.8	0.14 ± 0.05^∗∗∗^	0.14 ± 0.04^∗∗∗^	0.13 ± 0.04^∗∗∗^	24.26 ± 1.21	16.54 ± 1.1	23.84 ± 1.25	21.03 ± 0.81	10.02 ± 1.1	24.88 ± 2.49
	CS	12.07 ± 0.44	7.47 ± 0.32	9.9 ± 0.37	1.72 ± 0.02	1.41 ± 0.01	1.19 ± 0.01	29.7 ± 1.33	20.8 ± 2.08	21.4 ± 1.47	0.49 ± 0.03	0.21 ± 0.04^∗∗∗^	0.21 ± 0.04^∗∗∗^	26.31 ± 1.16	20.06 ± 1.17	21.73 ± 1.21	15.7 ± 1.16	17.18 ± 3.72	21.37 ± 1.53
	CF	39.72 ± 0.11^∗∗∗^	19.14 ± 0.17^∗∗^	21.94 ± 0.21^∗∗^	0.48 ± 0.08^∗∗^	0.34 ± 0.08^∗∗^	0.26 ± 0.05^∗∗^	25.8 ± 2.25	26.5 ± 1.89	33.4 ± 1.44	0.32 ± 0.02^∗∗^	1.65 ± 0.04	0.88 ± 0.03	44.91 ± 1.49^∗∗^	34.68 ± 1.19	37.36 ± 1.14^∗^	39.12 ± 4.91^∗^	38.57 ± 1.86^∗^	44.06 ± 4.04^∗^


*^a^CR, Codonopsis Radix; AMR, Atractylodis Macrocephalae Rhizoma; ASR, Angelicae Sinensis Radix; AsR, Astragali Radix; JF, Jujubae Fructus; GR, Ginseng Radix; AF, Amomi Fructus; AuR, Aucklandiae Radix; AC, Areca Catechu; DA, Dioscoreae Ahizoma; LSA, Lablab Semen Album; PC, Poria Cocos; NS, Nelumbinis Semen; CS, Coicis Semen; CF, Crataegi Fructus. ^*b*^GAE, gallic acid equivalent. ^*c*^W, water extract; E50, 50% ethanol extract; E90, 90% ethanol extract. ^*d*^Values are expressed as Mean ± SD, where *n* = 4, each with triplicate samples. Statistical comparison was made with mean value (mean value of total phenolics is 12.87 mg GAE/g; mean value of DPPH radical scavenging EC50 is 1.38 mg/mL; mean value of tBHP protection effect is 37.9%; mean value of tBHP protection effect EC50 is 0.65 mg/mL; mean value of inhibition effects to ROS formation is 30.5%; mean value of pARE-luciferase activity increase is 29.6%). ^∗^*p* < 0.05; ^∗∗^*p* < 0.01; ^∗∗∗^*p* < 0.001.*

The free radical scavenging activity of the polyphenolic compounds in herbal extracts was measured with DPPH radical scavenging assay. DPPH, having stable free radicals, was used to detect radical-scavenging ability of herbal extracts. Here, the EC50 of each herbal extract was determined (Figure 10). The DPPH radical scavenging activity of most extracts were similar (Figure 10 and Table 3). Among all the herbs, Lablab Semen Album showed the largest value of EC50, indicating the weakest anti-oxidant. In contrary, Arecae Pericarpium and Crataegi Fructus showed stronger DPPH radical scavenging activity: the outcome was consistent with the result of Folin-Ciocalteu assay (Figure 10).

To reveal the protective effect, the herbal extract was applied onto the tBHP-treated RAW264.7 macrophages. RAW264.7 murine cell was chosen because of its high breeding speed and stability. A stress inducer, tBHP, was chosen to damage the macrophage, which induced cell death in a dose-dependent manner. Vitamin C served as a positive control (Huang et al., 2018a,b). A series of concentrations was employed, and the 15 herbs were divided into four groups, according to their efficiency (Supplementary Figure 2). After the optimization of dose of herbal extracts with MTT assay, the protective effects of the extracts to tBHP-induced cell cytotoxicity were determined. As shown in Supplementary Figure 3, the extracts protected the cells from oxidative damage in a dose-dependent manner. The protective effects and EC50 of the herbal extracts were summarized in Figure 10 and Table 3. The extracts of Angelicae Sinensis Radix and Ginseng Radix possessed the best protection effects to more than 50%, as compared to the control. In contrast, Aucklandiae Radix, Nelumbinis Semen and Coicis Semen provided significantly lower protective effects at around 20% (Figure 10).

The formation of ROS is one of the vital causes in inducing cell death. By determining the inhibition effects of herbal extracts to tBHP-induced ROS formation, the protective effect of herbal extracts was further compared and analyzed. As shown in Table 3, the inhibition effects to ROS formation were positively related with the protective effects to cell viability. The 50% ethanol extracts of Atractylodis Macrocephalae Rhizoma, Angelicae Sinensis Radix, Jujubae Fructus possessed the best effect having over 50% of inhibition, as compared to the control (Figure 10). Moreover, the luciferase-reporter construct (i.e., pARE-Luc) containing four ARE DNA regulatory elements deriving from the promoters of anti-oxidative genes, tagged upstream of a luciferase gene, was transfected into cultured RAW264.7 cells. The activation of pARE-Luc, triggered by herbal extracts, showed the same pattern as the result of ROS formation. Again, the 50% ethanol extracts of Atractylodis Macrocephalae Rhizoma, Angelicae Sinensis Radix, Jujubae Fructus exhibited the best inhibition effects of over 50% (Figure 10 and Table 3).

### Correlation Between Yin-Yang Attributes With Anti-oxidative Profile

After establishment of anti-oxidative profiles of spleen-meridian herbs, the correlation of this activity with the “Yin-Yang” classification criterion was determined. By comparing the specific anti-oxidative data of herbal extracts with the mean value of 45 extracts, the statistical tests were performed by one-way ANOVA with multiple comparisons using Dunnett’s test (Figure 10 and Table 3). According to the comparison results, the differences between species were relatively higher than that from different herbal extracts. Moreover, the correlation between the anti-oxidative profiles of the spleen-meridian herbal extracts with the “Yin-Yang” classification criterion was rather weak in the tested parameters. Poria Cocos is a plain medicine used to fortify spleen, while Crataegi Fructus is classified as Yin herb due to its acid flavor. These herbs contain abundant phenolic compounds and possess appreciable anti-oxidative activity, resulting in better protective effects to oxidative stressed cells than that from Yang herbs, e.g., Amomi Fructus, Aucklandiae Radix (Figure 10).

**FIGURE 11:**
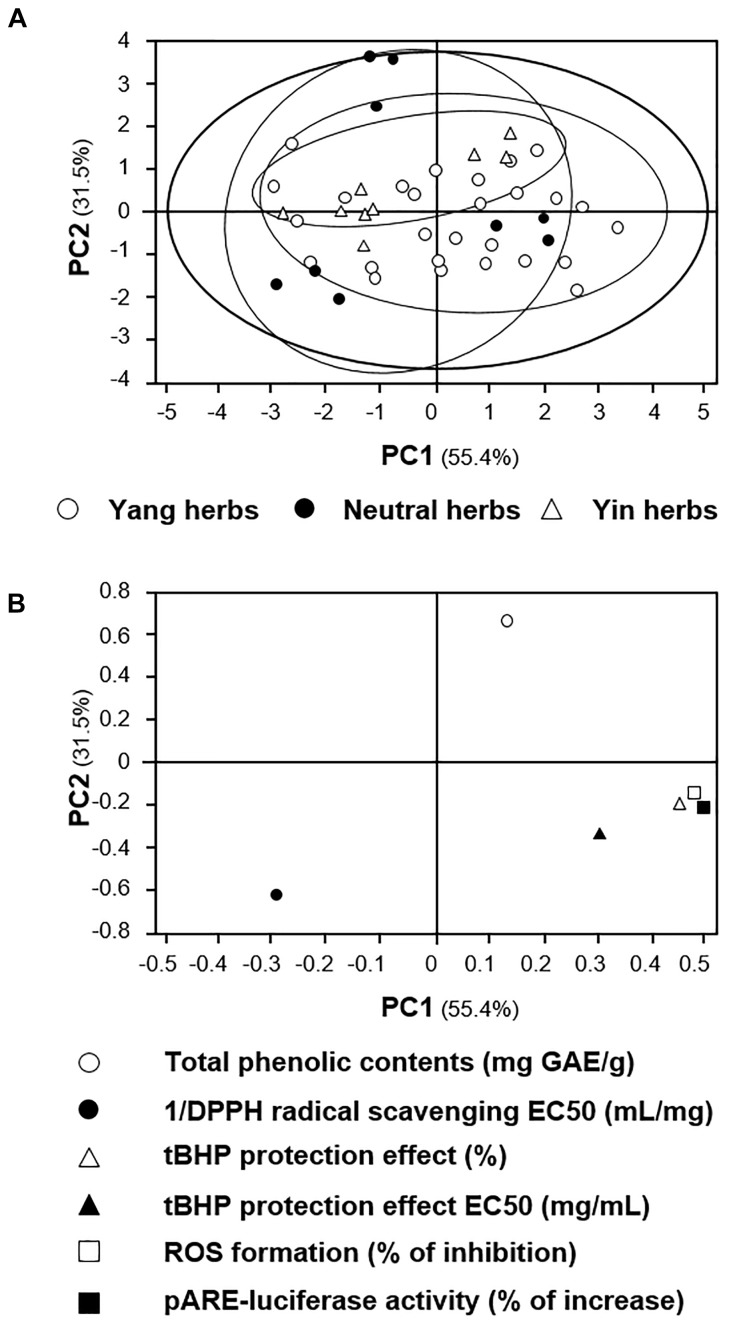
PCA analyses of anti-oxidative profiles of signals from the 15 spleen-meridian herbs. **(A)** Scoring plots. **(B)** Loading scatter plots. The distribution of each herbal extract in score scatter plots was calculated according to its correlation with the major components. The classification of the herbal extracts was according to the “Yin-Yang” nature of herbs, i.e., Yin-stimulating, neutral and Yang-stimulating herbs, according to TCM practice. The results showed low discrimination between herbal extracts with anti-oxidative properties. Each dot in the loading scatter plots **(B)** represents a variable, i.e., the parameters reflecting the anti-oxidant of extracts.

Furthermore, the PCA analysis of anti-oxidative profiles was conducted. As shown in Figure 11, the two principal components of the mass spectrum accounted for 86.9% of total variance, which is enough to support the credibility of the results. The distribution of Yang, neutral and Yin herbs could not be distinguished in the score scatter plot, indicating there are no significant difference between anti-oxidative profiles of these herbs (Figure 11A). This insignificant distinction could also be predicted from the loading scatter plot (Figure 11B). Having the parameters of tBHP-protected effect (%), ROS formation (% of inhibition) and pARE-luciferase activity (% of increase) at similar locations, there should be insufficient variables to distinguish the samples. Thus, the anti-oxidative properties of herbal extract were not equivalent to its “Yin-Yang” properties of herbal medicine.

## Discussion

The concept of four natures and five flavors of TCM herb is accumulated from long-term medical practice, and this theory plays a key role in clinical medication. To clarify the modern physiological meaning of the “Yin-Yang” properties, 15 spleen-meridian herbs were selected for the illustration of such classification. In accordance to TCM clinical practice, we have chosen different types of herbs that are known to have certain pharmacological functions: (i) herbs to fortify spleen and promote digestion, e.g., Aucklandiae Radix and Crataegi Fructus; (ii) herbs to keep digestion system in normal state by resolving dampness and treating diarrhea, e.g., Atractylodis Macrocephalae Rhizoma, Amomi Fructus, Areca Catechu, Dioscoreae Ahizoma, Lablab Semen Album, Nelumbinis Semen and Coicis Semen; (iii) herbs to replenish Qi and nourish blood, e.g., Codonopsis Radix, Angelicae Sinensis Radix, Astragali Radix, Jujubae Fructus and Ginseng Radix; and (iv) herbs to calm the heart and soothe the nerves, e.g., Poria Cocos and Nelumbinis Semen. Apart from the classification according to their pharmacological activity, these herbs could also be further divided into three groups, Yin, Yang and neutral herbs. Thus, the herbs chosen in present study is trying to cover the most commonly used TCM in treating spleen-related unwell.

Identifying chemical composition in herbal extract is the key to probe the properties of TCM herbs. However, the natural products always accompany with a huge amount of chemical variables, which greatly shake the credibility of any outcome (Kuang et al., 2012). Here, we employed standard methods, i.e., HPLC fingerprinting, NMR profiling and LC-MS profiling, as to reduce the variables in herbal extracts. Generally, HPLC fingerprinting is adopted to show all compounds with ultraviolet absorption; ^1^H-NMR profiling is able to display all compounds with active hydrogen; while LC-MS profiling is capable to show trace compounds (Zhao et al., 2005; Crockford et al., 2006). To obtain information of the components as much as possible, the detection wavelength of HPLC was set from 190 to 400 nm, and the gradient elution programs covered 0–100% acetonitrile. The chemical shift range was set from -2 to 12 ppm covering active hydrogen in almost all kinds of organic matters. Furthermore, the identified signals were subjected to full spectrum PCA determination, and which did not request the true nature of identified chemicals and could therefore reduce the possible variation (Savorani et al., 2010; Lu et al., 2014).

According to previous research, the herbal compounds associated with cold nature generally possess more polar structures; in contrast the compounds associated with hot nature have lower molecular weight; and the neutral compounds have a higher polar surface area (Fu et al., 2017a). The cold and hot properties of TCM are defined according to the impacts to human physiological condition, indicating the importance of chemical composition of herbal extracts. By using various analytic methods, we generated a large group of chemometrics data, and these data were subjected to PCA analysis. The results of LC-MS profile showed that the Yang-stimulating herbs contained more compounds of lower molecular weight and with less polar structures. This result is consistent with the previous proposed notion (Fu et al., 2017a). However, the results of HPLC and NMR profiles were not able to prove above conclusion, which might due to low resolution and similar chemical shift of most of the organic compounds.

Apart from the chemical basis, the differences of biological effects are always the most concern issues in studying the four natures of TCM herbs. Due to the high similarity of the opposition and interdependence relationship in “Yin-Yang” theory and redox system, the study on their connection was continuously. By using an oxygen radical absorbance capacity (ORAC) assay to compare the free radical-scavenging activity of Yin- and Yang-tonic herbs, Ou et al. (2003) was among the first groups to provide physical meaning of “Yin and Yang” in relating to biochemical processes. The result revealed that the amount of total phenolic compounds in Yin-tonic herbs were generally higher, and meanwhile which possessed a higher antioxidant activity than the Yang-tonic herbs. Thus, the authors deduced that Yin-tonic represents anti-oxidation, and Yang-tonic represents oxidation involved in energy metabolism. However, their result was against by Szeto and Benzie (2006): they measured the 1,1-diphenylpicrylhydrazyl radical-scavenging activity of Yin- and Yang-tonic herbs and found that most of the ‘Yang-invigorating’ herbs possess higher anti-oxidative activity. However, their conclusion was still not finalized due to different selection criterions, and the thoughtful analytic methods were not used. To set up a more credible and convincing antioxidant profiles, our present approach adopted various common assays at different levels, as to evaluate anti-oxidative properties of unknown herbal samples. Accompanied with PCA method, the current results showed that the correlation between “Yin-Yang” and anti-oxidative properties was relatively low, indicating the connection between these two systems might not be closely related, at least in ours 15 spleen-meridian herbs.

## Author Contributions

KT and TD conceived and designed the experiments. YH, LW, and PY performed the experiments, analyzed the results, and made figures and tables. KL, HW, XK, and YC contributed to scientific discussions. KT and YH wrote the paper.

## Conflict of Interest Statement

The authors declare that the research was conducted in the absence of any commercial or financial relationships that could be construed as a potential conflict of interest.
