# Supplementary material for: The Yin-Yang Property of Chinese Medicinal Herbs Relates to Chemical Composition but Not Anti-Oxidative Activity: An Illustration Using Spleen-Meridian Herbs
Source: Front Pharmacol. 2018 Nov 15;9:1304. doi: 10.3389/fphar.2018.01304 (PMC6249273; doi:10.3389/fphar.2018.01304)
Supplement: Supplementary file 1 [file Pesentation_1.pptx]

## Slide 1
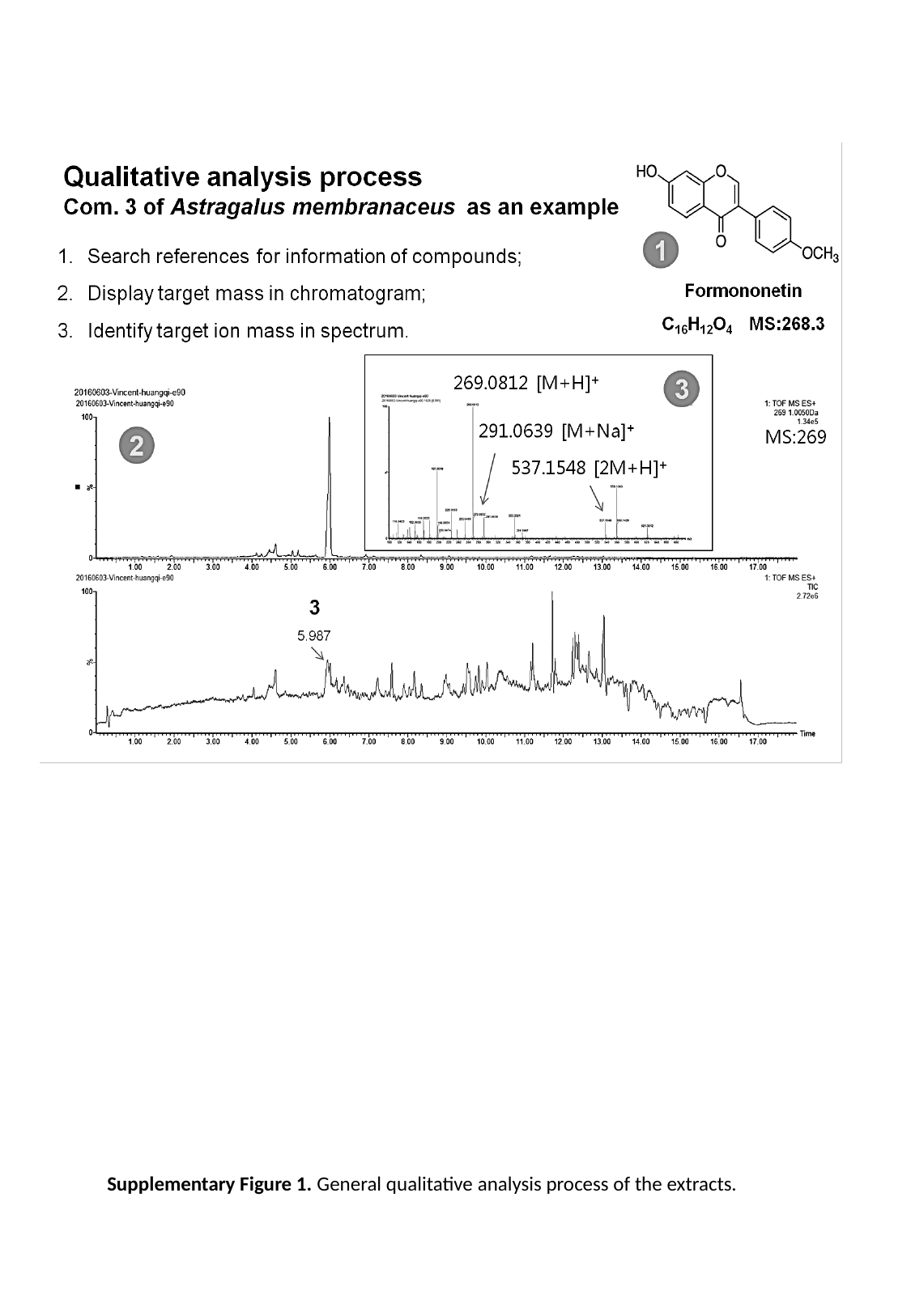

Supplementary Figure 1. General qualitative analysis process of the extracts.

## Slide 2
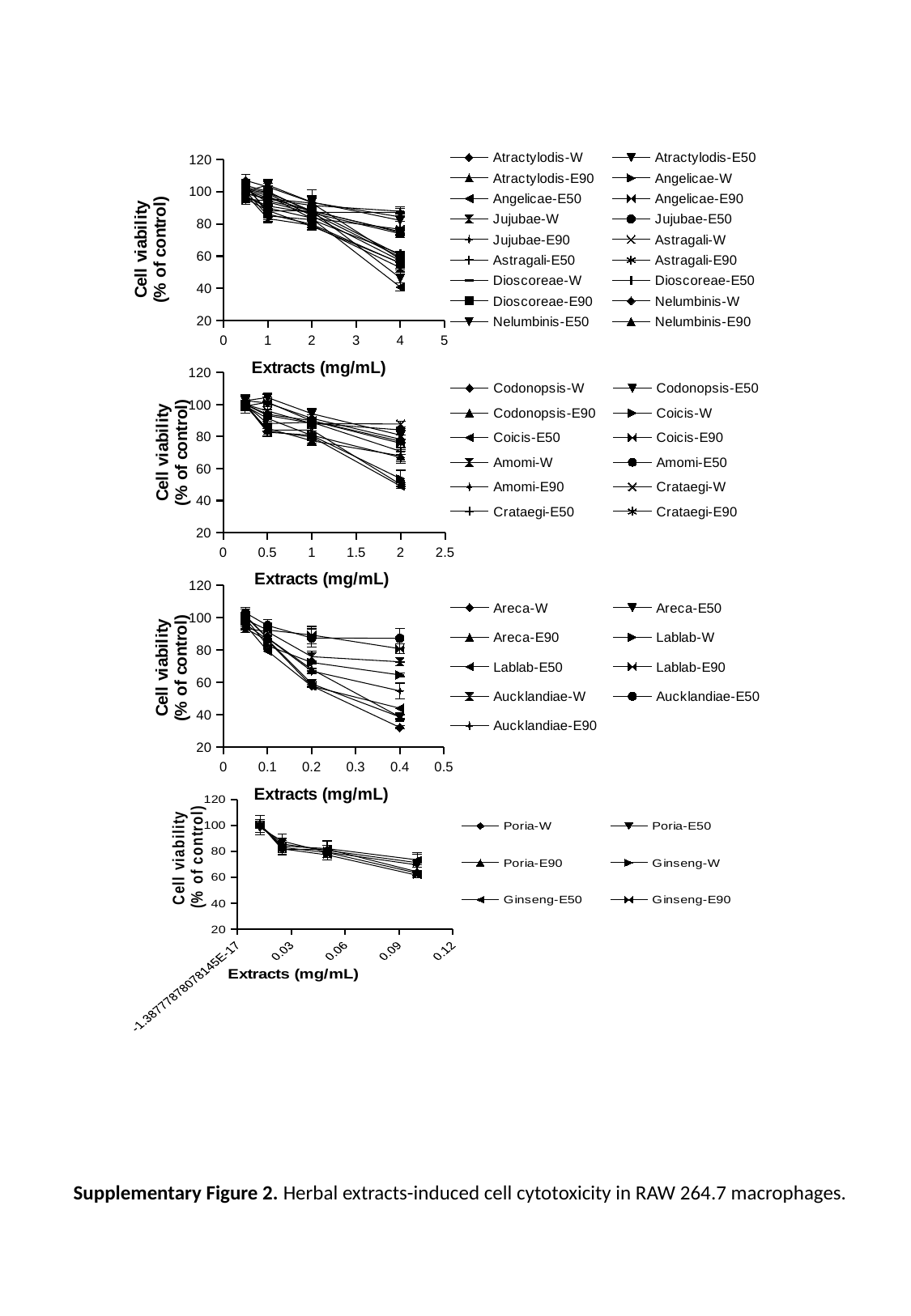

### Chart
| Category | Atractylodis-W | Atractylodis-E50 | Atractylodis-E90 | Angelicae-W | Angelicae-E50 | Angelicae-E90 | Jujubae-W | Jujubae-E50 | Jujubae-E90 | Astragali-W | Astragali-E50 | Astragali-E90 | Dioscoreae-W | Dioscoreae-E50 | Dioscoreae-E90 | Nelumbinis-W | Nelumbinis-E50 | Nelumbinis-E90 |
|---|---|---|---|---|---|---|---|---|---|---|---|---|---|---|---|---|---|---|
### Chart
| Category | Codonopsis-W | Codonopsis-E50 | Codonopsis-E90 | Coicis-W | Coicis-E50 | Coicis-E90 | Amomi-W | Amomi-E50 | Amomi-E90 | Crataegi-W | Crataegi-E50 | Crataegi-E90 |
|---|---|---|---|---|---|---|---|---|---|---|---|---|
### Chart
| Category | Areca-W | Areca-E50 | Areca-E90 | Lablab-W | Lablab-E50 | Lablab-E90 | Aucklandiae-W | Aucklandiae-E50 | Aucklandiae-E90 |
|---|---|---|---|---|---|---|---|---|---|
### Chart
| Category | Poria-W | Poria-E50 | Poria-E90 | Ginseng-W | Ginseng-E50 | Ginseng-E90 |
|---|---|---|---|---|---|---|Supplementary Figure 2. Herbal extracts-induced cell cytotoxicity in RAW 264.7 macrophages.

## Slide 3
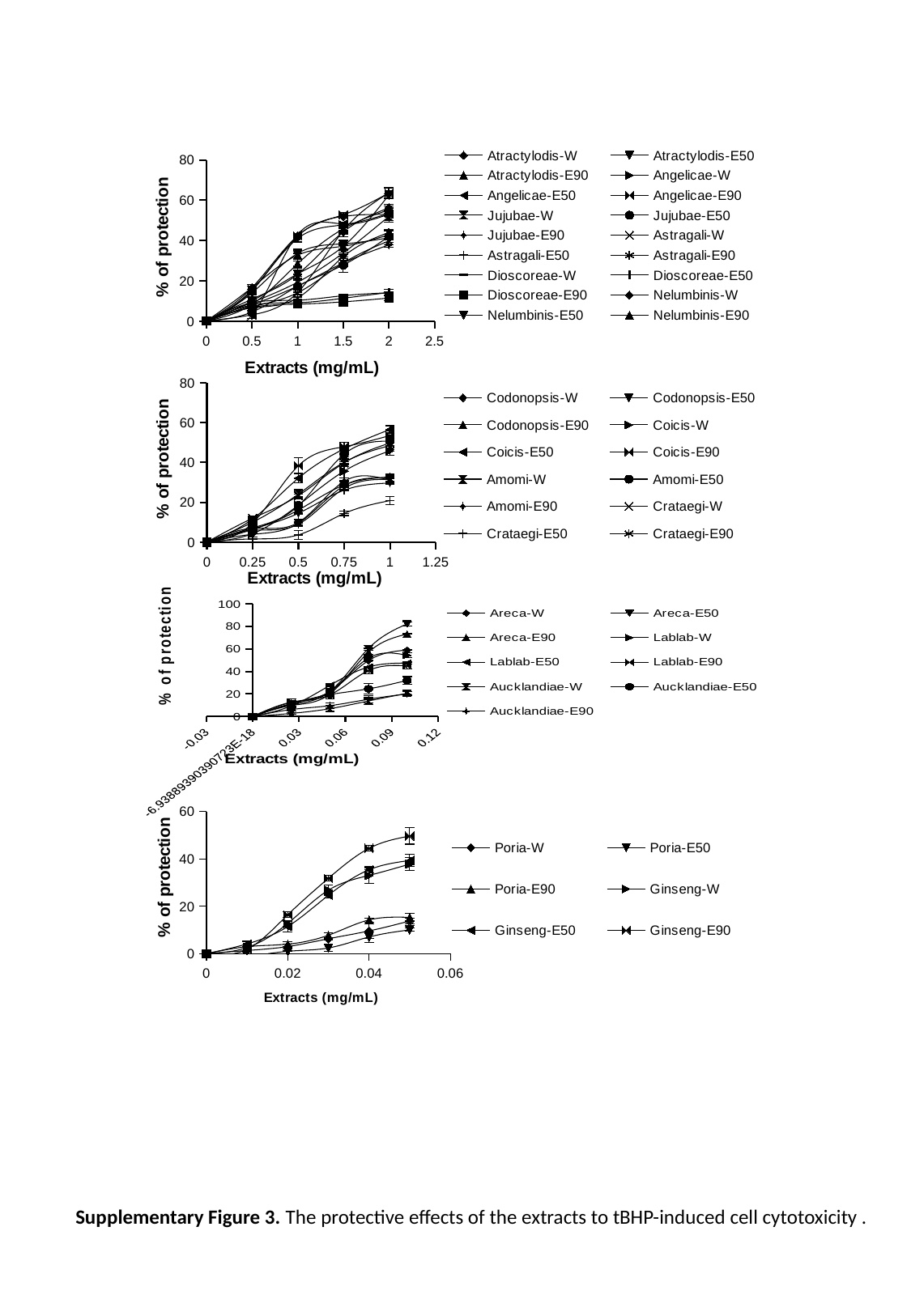

### Chart
| Category | Atractylodis-W | Atractylodis-E50 | Atractylodis-E90 | Angelicae-W | Angelicae-E50 | Angelicae-E90 | Jujubae-W | Jujubae-E50 | Jujubae-E90 | Astragali-W | Astragali-E50 | Astragali-E90 | Dioscoreae-W | Dioscoreae-E50 | Dioscoreae-E90 | Nelumbinis-W | Nelumbinis-E50 | Nelumbinis-E90 |
|---|---|---|---|---|---|---|---|---|---|---|---|---|---|---|---|---|---|---|
### Chart
| Category | Codonopsis-W | Codonopsis-E50 | Codonopsis-E90 | Coicis-W | Coicis-E50 | Coicis-E90 | Amomi-W | Amomi-E50 | Amomi-E90 | Crataegi-W | Crataegi-E50 | Crataegi-E90 |
|---|---|---|---|---|---|---|---|---|---|---|---|---|
### Chart
| Category | Areca-W | Areca-E50 | Areca-E90 | Lablab-W | Lablab-E50 | Lablab-E90 | Aucklandiae-W | Aucklandiae-E50 | Aucklandiae-E90 |
|---|---|---|---|---|---|---|---|---|---|
### Chart
| Category | Poria-W | Poria-E50 | Poria-E90 | Ginseng-W | Ginseng-E50 | Ginseng-E90 |
|---|---|---|---|---|---|---|Supplementary Figure 3. The protective effects of the extracts to tBHP-induced cell cytotoxicity .
